# Episodic future thinking and compassion reduce non-compliance urges regarding public health guidelines: a randomised controlled trial

**DOI:** 10.1186/s12889-023-15031-0

**Published:** 2023-01-28

**Authors:** Simon T. van Baal, Antonio Verdejo-García, Jakob Hohwy

**Affiliations:** 1 Cognition and Philosophy Lab, Monash University, 20 Chancellors Walk, Clayton, VIC 3800 Australia; 2 Department of Psychology, University of Warwick, Coventry, CV4 7AL UK; 3 Turner Institute for Brain and Mental Health, School of Psychological Sciences, Monash University, 18 Innovation Walk, Clayton, VIC 3800 Australia; 4 Monash Centre for Consciousness & Contemplative Studies, Monash University, 29 Ancora Imparo Way, Clayton, VIC 3800 Australia

**Keywords:** Impulsivity, Episodic future thinking, Compassion, Self-control, Urges, Pandemic

## Abstract

**Background:**

People often feel urges to engage in activities that violate pandemic public health guidelines. Research on these urges has been reliant on measures of typical behaviour, which fail to capture these urges as they unfold. Guideline adherence could be improved through interventions, but few methods allow for ecologically valid observation of the range of behaviours that pandemic guidelines prescribe.

**Methods:**

In this preregistered parallel randomised trial, 95 participants aged 18–65 from the UK were assigned to three groups using blinded block randomisation, and engaged in episodic future thinking (*n* = 33), compassion exercises (*n* = 31), or a control procedure (*n* = 31). Following an ecological momentary assessment procedure, participants report on the intensity of their occurrent urges (min. 1, max. 10) and their ability to control them. The study further investigates whether, and through which mechanism, state impulsivity and vaccine attitudes affect guideline adherence.

**Results:**

Episodic future thinking (*b* = -1.80) and compassion exercises (*b* = -1.45) reduced the intensity of urges. State impulsivity is associated with stronger urges, but we found no evidence that vaccine hesitancy predicts lesser self-control.

**Conclusions:**

We conclude that episodic future thinking exercises and compassion training may be used to decrease non-compliance urges of individuals who are an acute public health risk for the community, such as those in voluntary isolation.

**Supplementary Information:**

The online version contains supplementary material available at 10.1186/s12889-023-15031-0.

## Background

It is challenging to study predictors and methods for improving pandemic public health guideline adherence because such behaviour is not readily observed in laboratory settings, nor easy to reveal with self-report cross-sectional surveys. Using ecological momentary assessment rather than one-shot surveys, the focus of this study is to find whether episodic future thinking and compassion exercises could contribute to increasing adherence to public health guidelines for preventing COVID-19 spread. We also investigate whether state impulsivity and vaccine attitudes predict guideline adherence, while assessing through which mechanism these predictors affect behaviour.

Much research focuses on designing public health communication to achieve optimal public health guideline adherence [1]. Protective behaviours such as staying home during a lockdown can have immediate adverse impact on people’s financial situation [2], mental health [3–5], and physical health [6, 7]. In contrast, the effects of non-adherence are often less immediate: it may take time before symptoms from infection and accompanying negative consequences are experienced; there may be subsequent effects on others rather than oneself, such as by infecting loved ones or causing outbreaks in the community. Decisions on adopting protective behaviours therefore constitute a dilemma between choosing the long-term greater good versus the short-term individual gain. Here we test if increasing people’s future-orientedness and compassion can stimulate the adoption of protective behaviours during a pandemic.

To increase future-orientedness, we use episodic future thinking (EFT): imagining or simulating experiences that might occur in one’s future. EFT decreases the degree to which rewards are devalued if they are received further in the future, known as delay discounting [8, 9], which implies that the perceived value of immediate rewards will be diminished relative to future rewards. This means that EFT likely affects the intensity of urges, though the effects of EFT in various domains suggest that EFT might also impact self-control independently of the strength of urges [10–12].

Adopting protective behaviours is ultimately also prosocial, and prosocial behaviour can be enhanced by stimulating compassion, the feeling that arises in witnessing another’s suffering and that motivates a subsequent desire to help [13]. Compassion training has a valuation element in addition to a behavioural element, which means compassion training could affect both the intensity of urges and self-control [14, 15], both of which this study will investigate, in the context of urges of non-adherence to protective behaviours in a pandemic.

In addition, vaccine hesitancy, attitudes on the effectiveness of these vaccines, and predictions about how soon the pandemic will end could factor into people’s behaviour. These attitudes are usually linked to other attitudes and behaviours relevant to pandemic behaviour such as lesser social distancing and mask-wearing [16, 17]. We therefore also investigate how such predictors of guideline adherence influence moment-to-moment behaviour.

Impulsivity, the tendency to make rapid responses for short-term gratification and with insufficient regard for negative consequences [18], is negatively correlated to public health guidelines adherence [19, 20]. The excessive delay discounting characteristic of high impulsivity can be influenced by fluctuations in internal states [21]. To understand how impulsivity affects moment-to-moment behaviour, it is important to gauge people’s mental state when behaviours occur [22, 23]. Thus, we study these behaviours in an ecologically valid manner, where proximal information on state impulsivity is obtained.

This study seeks to avoid the distortions that often afflict self-report measures about typical behaviour [24]. To gain insights into moment-to-moment protective behaviour in the ‘wild’ and real-time changes following behaviour change interventions, we employ an ecological momentary assessment (EMA; or experience sampling) paradigm.

In our preregistered analyses, we predicted that both the compassion intervention and the episodic future thinking intervention would increase the likelihood of controlling urges, where we analyse their effect on self-control and the intensity of urges. Furthermore, we predicted that state impulsivity is associated with stronger urges and fewer attempts to resist urges, and that vaccine hesitancy and shorter predicted back-to-normal time frames are negatively correlated with the likelihood of controlling an urge through diminishing self-control.

## Methods

### Design

This study used a individually randomised parallel group trial with three groups: EFT, Compassion, and Control. In a single-blind procedure, participants were assigned to a group after they completed an eligibility assessment, which was also when baseline data was collected. By responding to survey prompts on their mobile phones, participants provided up to 5 repeated measurements throughout the day – maximally 35 surveys completed throughout the one-week long experiment. This study was preregistered https://osf.io/b5vxg/?view_only=dcb2b70d3e0148ca8d4929b6ea142ffd on 21/03/2021 on the Open Science Framework and registered as a clinical trial at clinicaltrials.gov on 02/09/2021, clinical trial ID: NCT05031559.

### Participants

The final sample contained 95 UK residents, recruited using volunteer sampling through the Prolific participant platform. Participants were 18–65 years of age, and within that age group, we created a representative sample based on sex and age (2 × 4), with age strata 18–29, 30–41, 42–53, 54–65, (e.g., 18–29-year-old females).

Participants (*n* = 293) completed an eligibility survey prior to the experiment, which was used to create a representative sample. Participants were added to the EMA software in two rounds in order to cope with varying drop-out across the eight demographic strata, since there was more dropout than anticipated during the EMA app download-phase.

Participants were asked whether they or one of their family members were part of a group that is vulnerable to COVID-19. Participants also answered questions on their willingness to take a COVID-19 vaccine and their beliefs about vaccine efficacy. We also elicited predictions of when people would be able to resume on-site work (insofar as that will ultimately be the case), when people would be able to go on holiday, and when life would go back to ‘normal’. These predictions and vaccination attitudes were then combined into three scores, varying from 0 to 10.

Notably, the UK government announced on 29 March that 6 people from 6 different households would be allowed to meet outside. This, together with Easter weekend, produced a situation wherein people were likely to have non-adherence urges.

### Randomisation and masking

Groups were assigned according to block randomisation (8 strata, 3 groups; sequence obtained from sealedenvelope.com), see Supplementary Materials for the distribution of age and sex per group. Participants were unaware of their condition assignment, but the experimenter was (considering that they had to be added to the appropriate group in the software). Participants were either assigned to the EFT condition, the compassion condition, or the control condition.

### Procedure

Each morning at 7.30am (expiry time 10am), participants would be asked to do either an EFT exercise, a compassion exercise, or reflect on a recent news story related to COVID-19. For the EFT exercise, participants were asked to imagine themselves in a future without COVID-19, for example, where they were travelling without restrictions. For the compassion exercise, participants were asked to imagine the suffering of individuals who were badly affected by COVID-19, for example, through the loss of family members. The news stories of COVID-19 concerned the negative impacts of COVID-19 on society (e.g., public health, business). All prompts are included in the Supplementary Materials. After each group-dependent prompt, participants would be prompted with “Remember that your behaviour has an effect on the COVID-19 situation”. Videos of the user interface are available.

Each day, after the morning survey, participants would receive 5 surveys that were available for 1 h. In randomised order, they were asked whether since the last survey they had felt an urge to *not* wash their hands, *not* cover their mouths when coughing or sneezing, *not* socially distance (e.g., to hug, shake hands), *not* leave details for contact tracing, or whether they had felt an urge to leave their house, touch their face, or avoid getting tested when it would have been better to do the opposite (from a COVID-19 standpoint). Participants responded using a slider [0,10], where 0 indicated no urge, 1 indicated a very weak urge, and 10 indicated a very strong urge. We then administered the Momentary Impulsivity Scale [25].

### Analysis

To determine sample size, we estimated an effect size of a 5-percentage point increase in the probability to control an urge in the EFT and Compassion groups, and assumed that participants would indicate they had an urge 3 times a day. We identified that 95% power, under these assumptions, could be achieved by collecting data from 90 participants. The power analysis is publicly available.

The intensity of urges was modelled using a cumulative link mixed model (CLMM) with a logit link, using the ‘ordinal’ R package [26]. The intensity of the urge was entered directly into the model—no averaging was conducted—and we included the following predictors: the group, the type of urge (and interaction between those), state impulsivity, with age, sex, the time of day and the day of the week as control variables, and the participant as the random intercept.

The type of urge was not included as a predictor in the main preregistered models, but it was specified in the exploratory analyses section and because the effects of the intervention might differ across domains, we decided to include it in the main analyses. In both analyses, we decided to deviate from the preregistration by including interaction terms between the type of urge and the intervention because we deemed it likely that the intervention might affect some urges more than others. The preregistration also specified the use of a linear mixed model, but due to the ordinal nature of the response variable, we deviated from this plan and conducted the analysis using a CLMM.

We used a binomial generalised linear mixed model (GLMM) to conduct the self-control analysis. In addition to the variables in the model above, vaccine hesitancy, vaccine effectiveness beliefs, back-to-normal timeline predictions, and whether participants attempted to resist the urge were included. The analysis was conducted using the ‘afex’ package [27].

A false discovery rate adjusted alpha of 0.05 was used to determine whether the effects based on the CLMM or on the GLMM were significantly different from those expected if the null hypothesis were correct. The ‘emmeans’ package [28] was used to conduct pairwise tests between factor levels; differences noted for the CLMM are on the latent scale (where the scale and location are arbitrary), while the differences for the GLMM are odds ratios. For numeric predictors, we zero-centered predictors to facilitate the interpretation of coefficients and differences between factor levels, and we used model coefficients to assess significance. For categorical predictors, we used sum contrast coding to compare the effects of independent variables against the grand mean. Effect sizes are reported as odds ratios. Effect sizes are reported as odds ratios.

Our analyses were preregistered, data and analysis are available [29].

## Results

The experiment took place from 29 March to 4 April 2021. The UK was in a state of lockdown then, but most regions in the UK were in the early phases of reopening. In total, 200 participants were added to the EMA software, 112 of whom downloaded the app, and 97 of those completed more than 50% of the EMA surveys. As indicated in our preregistration, participants who completed less than 50% of EMA surveys were excluded. Finally, two participants never reported having urges of non-adherence, and thus these were excluded from the final sample. In the final sample, there were 40 (42.1%) males, with a mean age of 41.0 (SD = 14.0), and 55 females (57.9%) with a mean age of 41.0 (SD = 12.5). See Table 1 for demographic information per group; see Figure S2 for a CONSORT diagram describing the flow of the recruitment process.

**Table 1:** Demographic information of the sample, per group

Condition	Mean Age (SD)	Male	Vaccinated	Covid Diagnosis
Compassion	41.8 (13.5)	35.5%	41.9%	6.5%
Control	39.8 (13.2)	41.9%	45.2%	3.2%
EFT	41.8 (13)	48.5%	39.4%	6.1%

Further, 40 participants (42.1%) reported they had received a COVID-19 vaccine, and 5 reported that they had been diagnosed with COVID-19 at some point, with one participant reporting they had experienced both of these events. Even though the individual risk of COVID-19 is mitigated for vaccinated individuals and those who previously contracted COVID-19, they were still required to comply with the guidelines for various reasons. Therefore, vaccinated individuals were not excluded.

Missing data in EMA study designs usually occurs survey-wise [30], which is also the case in our study—8.7% of the data collection surveys were missing in the final sample, with only 0.9% of the data collection surveys missing individual values. Many participants missed surveys on Monday because they were still familiarising with the software. Missing data for the data collection surveys was disproportionately concentrated in the responses of female participants (10.2% missing data for females; 7.3% for males). Moreover, participants with missing data tended to be slightly older (*M*_age_ = 43.7 for missing data entries; *M*_*age*_ = 40.2 for non-missing data entries).

Without the access to other momentary information that correlates with the outcome variables, this pattern of missingness largely prohibits the use of modern data methods to eliminate bias or improve statistical power. We did not find evidence the day of the week, the sex or age of participants impacted the outcome variables, so there appears to be no need to assume the pattern of missingness in this study gives reason to be concerned about biased estimates. Therefore, we conclude that the data is missing at random (MAR), and these variables are included in the statistical models so the risk of biased estimates is limited [31]. The other 1.1% of surveys containing missing data only missed values on the MIS, most likely due to a software error. We found no observable patterns in the other variables for these missing values, so here too we will assume these data are MAR.

Different types of urges occurred at different rates: over the one-week-long experiment, 83 out of the 95 participants reported the urge to leave the house at least once, and did so 6.80 times on average (*SD* = 6.09), 79 reported the urge to touch their face (*M* = 7.91, *SD* = 8.14), 73 Participants reported the urge to disregard social distancing guidelines (*M* = 4.66, *SD* = 4.37, while 60 reported the urge to not wash their hands (*M* = 5.03, *SD* = 6.11). Only 33 participants reported the urge to not cover their mouth (*M* = 2.82, *SD* = 4.39), 21 participants reported the urge to not leave their contact details (*M* = 3.86, *SD* = 7.18), and 17 participants reported the urge to avoid getting tested (*M* = 4.82, *SD* = 7.77).

There was high variance in the number of urges people experienced, and the average number of urges experienced was similar over the different groups: in the EFT group, people had 20.0 urges on average (*SD* = 30.2) in the Compassion group, people had 23.1 urges on average (*SD* = 16.3) and 23.0 (*SD* = 26.7) in the Control group.

The various types of urges were different in their intensity and in their controllability. We report these differences partitioned by group in Table 2, but we also report general differences in the Supplementary Materials.

**Table 2:** The intensity of urges and the probability that participants controlled them, by allocation group

Type of Urge	Condition	Urge Intensity	Prob. Control
Not Covering Mouth	EFT	2.11 (0.92)	0.77 (0.92)
	Compassion	2.72 (1.3)	0.7 (1.3)
	Control	1.85 (0.43)	0.78 (0.43)
Leaving Home	EFT	4.13 (2.18)	0.45 (2.18)
	Compassion	4.25 (1.89)	0.44 (1.89)
	Control	5.06 (1.55)	0.43 (1.55)
Skip Leaving Contact Details	EFT	1.31 (0.66)	0.8 (0.66)
	Compassion	1.78 (0.62)	0.52 (0.62)
	Control	4 (0.94)	0.5 (0.94)
Not Socially Distance	EFT	4.34 (1.86)	0.57 (1.86)
	Compassion	3.6 (1.63)	0.67 (1.63)
	Control	3.46 (1.58)	0.74 (1.58)
Not Getting Tested when Experiencing Symptoms	EFT	1.58 (1.28)	0.21 (1.28)
	Compassion	2.02 (1.09)	0.2 (1.09)
	Control	5.83 (0.38)	0.12 (0.38)
Touching Face	EFT	2.93 (1.55)	0.27 (1.55)
	Compassion	3.27 (1.43)	0.39 (1.43)
	Control	3.45 (1.59)	0.51 (1.59)
Skip Washing Hands	EFT	2.74 (1.46)	0.77 (1.46)
	Compassion	2.81 (1.43)	0.75 (1.43)
	Control	3.22 (1.3)	0.72 (1.3)

### Predictors for urge intensity

**Fig. 1:**
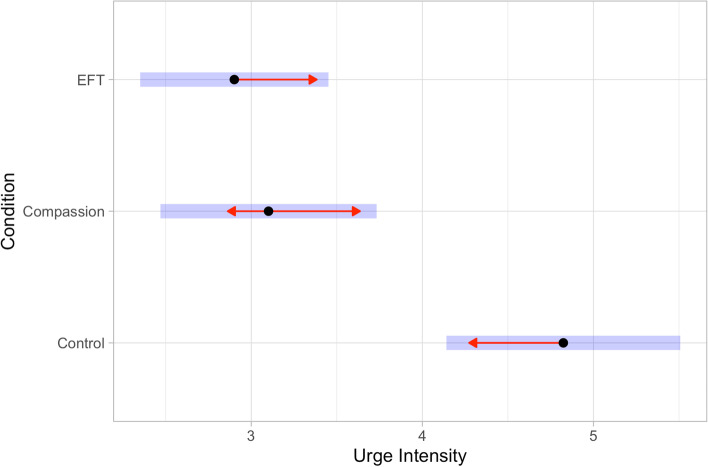
The effects of the between-participants conditions: Episodic Future Thinking manipulation (EFT; top of figure), the Compassion manipulation (middle), as compared to the Control condition (bottom), on the perceived intensity of urges (x-axis, location and scale are arbitrary). The points are estimated marginal means, the red arrows are comparison arrows reflecting the pairwise tests, and the error bars are 95% CIs

Participants experienced weaker urges in the EFT group, *b* = -1.798, 95% CI [-2.923, -0.672], *z* = -3.824, *p* < 0.001, and in the Compassion group, *b* = -1.449, 95% CI [-2.580, -0.317], *z* = -3.064, *p* < 0.01, than in the Control group. This means that, for instance, the average predicted odds of reporting a stronger urge in the Control condition than in the EFT condition across all urges, time points, and days was 1.798:1. Urge intensity was not significantly different in the EFT group from the Compassion group (*z* = 0.742, *p* = 0.46). See Fig. 1.

**Fig. 2:**
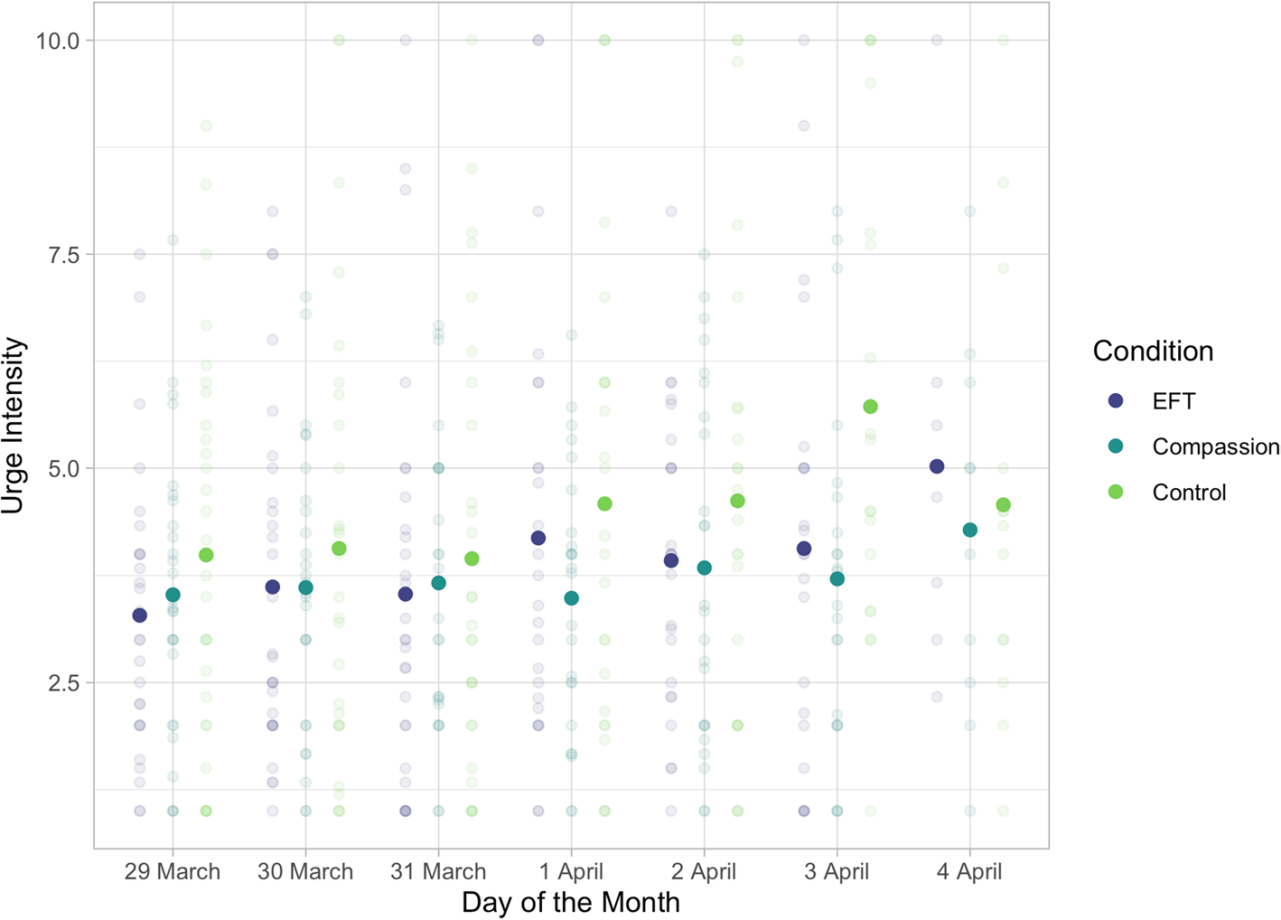
The intensity of urges (y-axis), partitioned by day (x-axis), and group (colour). The experiment was partially conducted over a public holiday, at which time stronger urges of non-adherence would be expected. 2 April (third line from the right) was Good Friday, and 4 April (the right-most line) was Easter Sunday. The coloured points represent the estimated marginal means, and the error bars are 95% CIs. The grey data points each represent the aggregated data of one participant

There were also interactions between group and type of urge: urges to avoid leaving details for contact tracing were weaker in the EFT group, *b* = -5.737, 95% CI [-7.598, -3.876]; *z* = 7.025, *p* < 0.0001, and in the Compassion group, *b* = -4.871, 95% CI [-6.776, -2.965]; *z* = 6.161, *p* < 0.0001, than in the Control group, but were not significantly different from each other, *z* = 1.021, *p* = 0.31. Further, urges to avoid getting tested were also weaker in the EFT group, *b* = -6.833, 95% CI [-8.879, -4.787]; *z* = 7.995, *p* < 0.0001, and in the Compassion group, *b* = -5.384, 95% CI [-7.396, -3.372]; *z* = 6.407, *p* < 0.0001, than in the Control group, but were not significantly different from each other, *b* = 1.449, 95% CI [-0.403, 3.302], *z* = 1.873, *p* = 0.06. See Fig. 2.

**Fig. 3:**
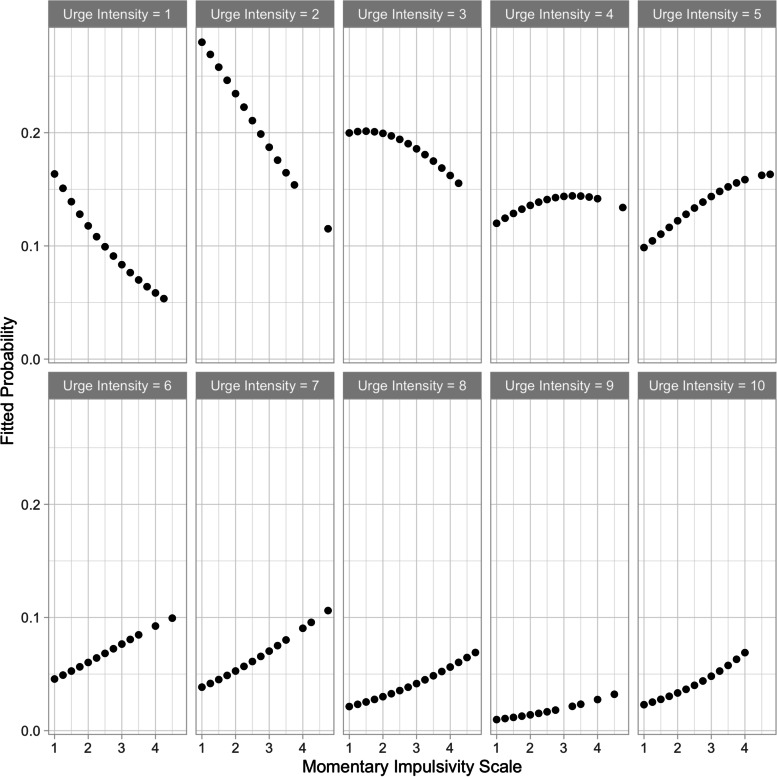
Fitted probabilities of a univariate cumulative link mixed model showing the relationship between the intensity of urges and scores on the Momentary Impulsivity Scale. The model assigns low probabilities to low-impulsivity, high-intensity combinations and high-impulsivity, low-intensity combinations

State impulsivity had a significant effect on the intensity of urges, *b* = 0.362 95% CI [0.207, 0.518], *z* = 4.580, *p* < 0.001. See Fig. 3 for a depiction of the relationship between state impulsivity and the intensity of urges.

### Predictors for self-control

There were no significant differences in self-control between the groups, all *ps* > 0.1.

There were also interactions between the type of urge and the group: people were significantly more likely suppress the urge to not cover their mouth when coughing or sneezing in the Control group than in the Compassion group, OR = 16.613, 95% CI [1.56, 177.58], *z* = 2.832, *p* = 0.01, but not than in the EFT group, OR = 3.403, 95% CI [0.24, 47.57], *z* = 1.087, *p* = 0.27. People were also more likely to suppress the urge to touch their face in the Control group than in the Compassion group, OR = 4.085, 95% CI [1.47, 11.39], *z* = 3.286, *p* < 0.01, and in the EFT group, OR = 3.198, 95% CI [1.12, 9.15], *z* = 2.648, *p* = 0.01.

State impulsivity did not have a significant effect on self-control, *b* = 0.045, 95% CI [-0.293, 0.203], *z* = 0.0356, *p* = 0.72. Furthermore, state impulsivity did not have a significant effect on the probability of attempting to resist an urge *b = *-0.103, 95% CI [-0.320, 0.114], *z* -0.928, *p* = 0.35.

Neither vaccine hesitancy nor judgments about vaccine effectiveness significantly predicted self-control, *b* = -0.098, 95% CI [-0.262, 0.066], *z* = -1.173, *p* = 0.24; *b* = 0.038, 95% CI [-0.213, 0.229], *z* = 0.070, *p* = 0.94. Neither did predictions about when life would go back to ‘normal’ after the pandemic, *b* = -0.141, 95% CI [-0.726, 0.483]; *z* = -0.393, *p* = 0.69.

## Conclusion

This study recorded the intensity and controllability of various types of urges pertinent to pandemic management. We were able to measure people’s urges of non-adherence to protective behaviours during a pandemic without risking recall bias, by using ecological momentary assessment. Our findings show that episodic future thinking and compassion exercises reduced the intensity of urges to avoid protective behaviours, but did not affect self-control. We also found that different urges occur at widely varying rates within and between participants, which is an important consideration when assessing the relative impact of these urges.

Our findings show that episodic future thinking and compassion exercises reduced the intensity of certain urges, but we found no evidence that it affected self-control in our sample. This finding broadly aligns with the evidence that EFT can enhance future-oriented decision making in various contexts [10–12]. Given that urges usually pertain to immediate rewards, this reduction in the strength of urges after an EFT exercise is most likely because EFT reduces the relative value of immediate rewards compared to future rewards [8, 9].

The mechanisms through which EFT and compassion exercises affect behaviour differ: EFT enhances future-oriented decision making in various contexts [10–12], supposedly by decreasing delay discounting [8]; compassion exercises can increase prosociality [13], and there is also some evidence it might also increase future-oriented decision making [32]. These mechanisms both likely promote the salience of the potential negative consequences to people’s actions, which may be the reason for their effectiveness in this context. Alternative explanations include that compassion exercises can lead to increased positive affect and motivation [33], and can be helpful to deal with daily stressors [34], which may also decrease the perceived intensity of urges.

These results suggest that an invitation to engage in EFT and compassion-inducing talking points could be incorporated into press conferences and some public announcements to decrease urges of noncompliance during public health crises. Additionally, people who pose a specific risk to the community (e.g., those in voluntary self-isolation after travelling abroad) could be invited to periodically perform such a task.

We found no evidence that vaccine attitudes or predictions of back-to-normal timelines were associated with self-control in our sample. Other studies show that vaccine hesitancy is correlated with lesser social distancing and mask wearing [17], but most studies reporting these relationships rely on judgements about typical behaviour, or intentions to comply with guidelines. The lack of evidence for a significant relationship between vaccine attitudes and guideline adherence in this study suggests that more research is needed to understand how these attitudes affect moment-to-moment behaviour.

Different urges occurred at varying rates within and between participants, which is an important consideration when assessing the relative impact of these urges. If an urge is relatively rare, but difficult to control, then it may not be as relevant for policymakers and other key stakeholders to see if the probability to control this urge can be increased. Urges to avoid getting tested, or to not leave contact details were relatively infrequent, but the fact that around 20% of the sample reported one of these urges at least once is worrying given their importance to pandemic management [35].

State impulsivity was related to stronger urges, but not to diminished self-control. This evidence suggests that state fluctuations in impulsivity play an important, but poorly understood role in determining public health guideline adherence during pandemics. A recent study has found this ‘bottom-up’ effect of state impulsivity for a different, more general domain of urges [36]. It also suggests that interventions targeting the internal state of the individual, and impulsivity in particular, might be effective at ameliorating their guideline adherence. Future research could investigate whether state impulsivity, as well as other internal states, can be targeted to improve public health guideline adherence.

### Limitations and future directions

The main limitations of this study were that the heterogeneity of the experience of certain types of urges rendered the sample size too small to draw accurate inferences in some domains. Only around 20 individuals, spread over three groups, reported having urges to avoid getting tested or to avoid leaving details for contact tracing at least once. Furthermore, the lack of evidence that the manipulations affected self-control could be due to a lack of power, rather than the absence of a meaningful effect. The assumptions we made for our power analysis were optimistic, especially because we did not account for participant clustering of responses.

The lack of power is also visible in the effect size estimates of the compassion manipulation’s influence on the likelihood of covering one’s nose and mouth while coughing or sneezing, where the spread of the confidence interval suggests near empty strata. Hence there remains considerable uncertainty about the effectiveness of the intervention in these domains.

This speaks to the strengths and weaknesses of the ecological momentary assessment paradigm because, on the one hand, it is a powerful paradigm for events that occur often and to a wide range of people (such as the urge to abandon social distancing), and it can provide insight into behaviour ‘in the wild’. On the other hand, for events that only happen for a narrow subset of people, or that happen infrequently, ecological momentary assessment needs to be applied to that particular subset, or another approach should be considered. A further limitation concerns the extent to which the findings apply to different populations. Ecological momentary assessment is known to generate missing data, which creates uncertainty about effect size estimates and reduces overall data quality. In our study, we mitigated this cost of ecological approaches by incentivising high compliance and implementing a compliance threshold. Nonetheless, the effect size estimates we report should be considered in light of the uncertainty associated with missing data.

Another limitation of the current work is that we did not administer any baseline scales to investigate whether individual differences, such as impulsivity or compassion traits, predict responsiveness to the interventions. The lack of evidence for a relationship between the baseline variables we collected and self-control also need not generalise to the population because of our sample size limitations.

In the current work, participants were asked to engage with others’ suffering and prompted to consider that their actions influence the situation they were asked to picture. We did not explore exactly how participants’ states changed after the manipulations to avoid demand characteristics, so we cannot be sure about the exact mechanisms that caused the observed effects. Future research could, for example, investigate whether only empathising with the suffering would be sufficient, or whether stronger language linking one’s own behaviour to others’ suffering would be more effective.

Future research could focus on the role of behavioural science interventions, including, but not limited to, episodic future thinking and compassion-inducing exercises, in producing desired behaviour during public health crises. In this line of research, it may be promising to also consider the combination of compassion and episodic future thinking, to see if the effects might interact positively.

Further research could also address whether individual differences can predict responsiveness to this type of intervention. We did not administer any baseline scales to investigate whether individual differences, such as impulsivity or compassion traits, predict responsiveness to the interventions.

We also deem it important that more research is devoted to uncovering the factors predicting moment-to-moment decision-making in people’s daily lives, where ecological momentary assessment and GPS data [37] could play a critical role. These methods can provide a more comprehensive understanding of the effects of behavioural interventions, shedding light on their longevity and externalities.

## Supplementary Information

**Additional file 1:**

## Data Availability

The datasets generated and/or analysed during the current study are available in the OSF repository, https://osf.io/pj9ft/.

## Abbreviations

EFT: Episodic future thinking
COVID-19: Coronavirus Disease 2019
LMM: Linear Mixed Model
GLMM: Generalised Linear Mixed Model
EMM: Estimated marginal mean
GPS: Global Positioning System
