# Supplementary material for: Episodic future thinking and compassion reduce non-compliance urges regarding public health guidelines: a randomised controlled trial
Source: BMC Public Health. 2023 Jan 28;23:189. doi: 10.1186/s12889-023-15031-0 (PMC9883827; doi:10.1186/s12889-023-15031-0)
Supplement: Supplementary file 1 — Additional file 1: [file 12889_2023_15031_MOESM1_ESM.docx]

Supplementary Materials

# Intensity and controllability of the types of urges

Reported urges, in order of intensity, were: leaving the house (M = 4.46, SD = 1.86), not socially distancing (M = 3.79, SD = 1.69), touching one’s face (M = 3.22, SD = 1.52), avoiding getting tested (M = 2.74, SD = .93), avoiding washing hands (M = 2.92, SD = 1.39), not leaving details for contact tracing (M = 2.60, SD = .83), and, weakest, not covering mouth and nose when coughing/sneezing (M = 2.23, SD = .93). For pairwise comparisons, see Figure 3.

Reported success at controlling urges, in descending order of likelihood, was: not washing hands (M = .748, SD = .074); not covering their mouths (M = .747, SD = .057); not socially distancing (M = .662, SD = .076); not leaving contact details for contact tracing purposes (M = .581, SD = .008); leaving the house (M = .441, SD = .102); touching one’s face (M = .390, SD = .095); avoiding getting tested (M = .186, SD = .038). See Figure 3. It is important to note that these probabilities must be judged together with the relative frequencies of each urge. For instance, the probability of controlling an urge to avoid getting tested is extremely low, but these urges were infrequently experienced.

Differing dropout across groups after the second round of participant entries led to an imbalance in the female/male sample division. Here we display the age and sex distribution between groups (Figure S1).


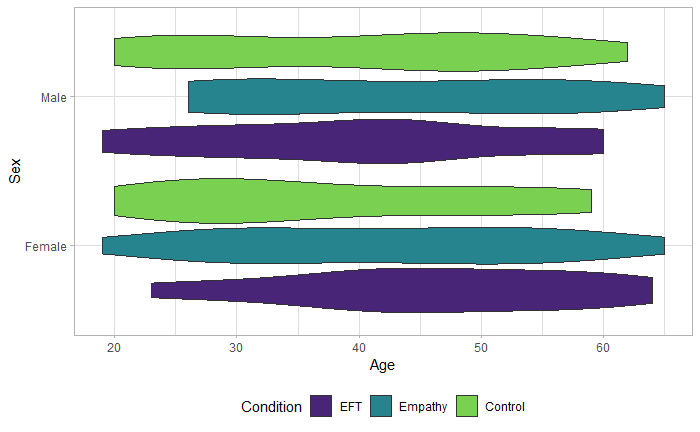


**Figure S1. The age and sex distribution of the sample.**

Below we also provide an adjusted CONSORT diagram to show the experimental procedure and the exclusions, see Figure S2. This diagram deviates from the standard CONSORT flow diagram because the design we used for the study differs substantially from the standard design in the medical sciences.


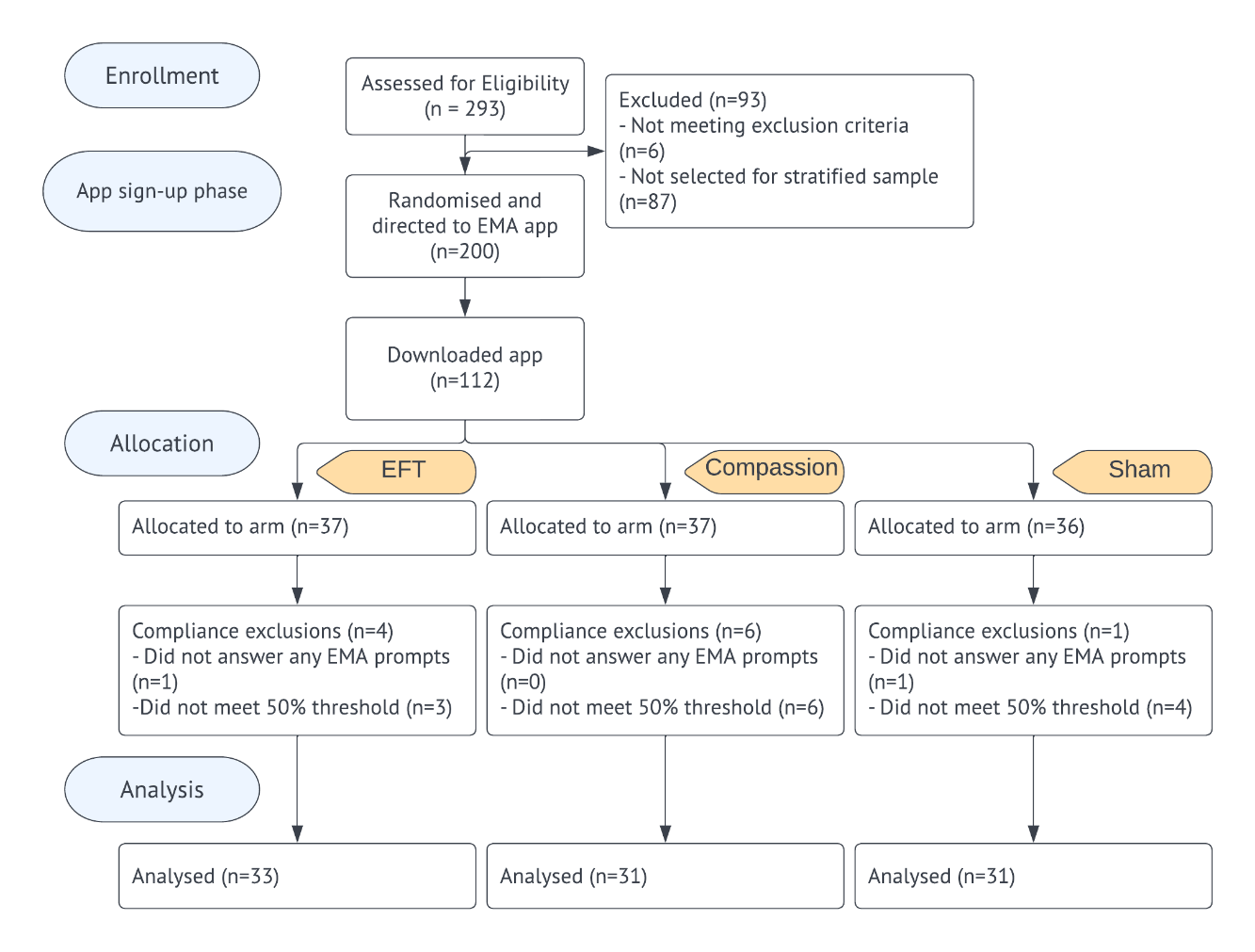


Figures S2. A consort diagram of the experimental procedure.

We also include all the prompts shown to the participants (see Table S1).

*Table S1. The instructional prompts participants received each morning in their 7.30am survey.*

| **Episodic future thinking** | **Compassion** | **Control** |
| --- | --- | --- |
| Please imagine yourself after lockdowns and restrictions are over. You are on holiday, at your favourite destination. Try to imagine how you feel, and picture your surroundings (think about smell and sounds too). | Please imagine yourself as a vulnerable person. You have severe asthma and will have significant trouble breathing if you catch COVID-19. Try to feel the emotions that might be going through you. | Please reflect on the news that shutdowns of the Ferguson shipyard during the Covid pandemic have added an extra £4.3m to the cost of two over-budget and delayed CalMac ferries. |
| Please imagine yourself in the future when lockdowns and restrictions are over. You are travelling to a place you always wanted to go. Imagine how you feel and picture your surroundings (think about smells and sound too). | Please imagine yourself as a family member of a person in hospital due to COVID-19. You want to be with them, but you are not allowed to because you might catch it yourself. Try to feel the emotions that might be going through you. | Please reflect on the news that the COVID-19 restrictions in Scotland were relaxed on 13 March. Up to four people from two different households can now gather outside. |
| Please imagine yourself in the future when lockdowns and restrictions are over, and picture that you are doing something you love. Imagine how you feel and picture your surroundings (think about smell and sound too). | Please imagine yourself as a vulnerable person. You are an elderly person in a retirement home. You have not been able to receive any visitors for months, and your interactions with the other residents are restricted. Try to feel the emotions that might be going through you. | Please reflect on the fact that on 5 March, COVID-19 infections were rising in Europe again. |
| Please imagine yourself after lockdowns and restrictions are over. You are doing your favourite activity that became possible after lockdowns lifted. Imagine how you feel, and picture your surroundings (think about smell and sound too). | Please imagine yourself as a healthcare worker in the emergency room. You have not been able to get proper sleep and have had to keep families separated because it was too dangerous to see their loved ones. Try to feel the emotions that might be going through you. | Please reflect on the reporting from 14 March that many people in Lincolnshire are not turning up for their vaccinations. |
|  |  | Please reflect on the news that on 14 March people were fined 800 pounds each for having a party in Gloucester. |
|  |  | Please reflect on the news that 48,000 businesses have signed up for workplace testing. |
|  |  | Please reflect on the news that 28% of people in need of social care have seen their health decline during COVID-19. |

*Table S2. Data structure information. This table shows the number of observations of variables segmented by whether an urge was reported by the participant or not. It also provides information about missing values. When a variable is numeric, the information provided in each cell is: mean (standard deviation); when it is a factor the information provided is: count (percentage).*

| **Label** | **Total N** | **Missing N** | **Levels** | **Urge not reported** | **Urge Reported** | **(Missing)** | **Total** |
| --- | --- | --- | --- | --- | --- | --- | --- |
| Total N (%) |  |  |  | 17770 (81.7) | 2092 (9.6) | 1894 (8.7) | 21756 |
| Urge magnitude | 2092 (10.5) | 17770 | Mean (SD) | NaN (NA) | 4.2 (2.7) | NaN (NA) | 4.2 (2.7) |
| Urge control | 2087 (10.5) | 17775 | Urge controlled | 0 (0.0) | 967 (46.2) | 0 (0.0) | 967 (4.4) |
|  |  |  | Urge not controlled | 0 (0.0) | 1120 (53.5) | 0 (0.0) | 1120 (5.1) |
|  |  |  | (Missing) | 17770 (100.0) | 5 (0.2) | 1894 (100.0) | 19669 (90.4) |
| MIS | 19683 (99.1) | 179 | Mean (SD) | 1.5 (0.6) | 1.8 (0.7) | 1.2 (NA) | 1.5 (0.7) |
| Type of urge | 19862 (100.0) | 0 | Cover mouth and nose | 2746 (15.5) | 93 (4.4) | 269 (14.2) | 3108 (14.3) |
|  |  |  | Leave your house | 2271 (12.8) | 565 (27.0) | 272 (14.4) | 3108 (14.3) |
|  |  |  | No contact tracing details | 2753 (15.5) | 84 (4.0) | 271 (14.3) | 3108 (14.3) |
|  |  |  | Not physical distancing | 2496 (14.0) | 340 (16.3) | 272 (14.4) | 3108 (14.3) |
|  |  |  | Not getting tested | 2754 (15.5) | 83 (4.0) | 271 (14.3) | 3108 (14.3) |
|  |  |  | Touch mouth or nose | 2215 (12.5) | 625 (29.9) | 268 (14.1) | 3108 (14.3) |
|  |  |  | Not washing hands | 2535 (14.3) | 302 (14.4) | 271 (14.3) | 3108 (14.3) |
| Time of day | 19862 (100.0) | 0 | Mean (SD) | 15.1 (3.5) | 14.8 (3.4) | 15.0 (3.8) | 15.0 (3.5) |
| Weekday | 19862 (100.0) | 0 | Mon | 1093 (6.2) | 151 (7.2) | 16 (0.8) | 1260 (5.8) |
|  |  |  | Tue | 2707 (15.2) | 431 (20.6) | 12 (0.6) | 3150 (14.5) |
|  |  |  | Wed | 2717 (15.3) | 344 (16.4) | 26 (1.4) | 3087 (14.2) |
|  |  |  | Thu | 2804 (15.8) | 331 (15.8) | 22 (1.2) | 3157 (14.5) |
|  |  |  | Fri | 2800 (15.8) | 305 (14.6) | 17 (0.9) | 3122 (14.4) |
|  |  |  | Sat | 2813 (15.8) | 265 (12.7) | 23 (1.2) | 3101 (14.3) |
|  |  |  | Sun | 2836 (16.0) | 265 (12.7) | 7 (0.4) | 3108 (14.3) |
|  |  |  | (Missing) | 0 (0.0) | 0 (0.0) | 1771 (93.5) | 1771 (8.1) |
| Sex | 19862 (100.0) | 0 | Female | 9990 (56.2) | 1128 (53.9) | 1230 (64.9) | 12348 (56.8) |
|  |  |  | Male | 7780 (43.8) | 964 (46.1) | 664 (35.1) | 9408 (43.2) |
| Age | 19862 (100.0) | 0 | Mean (SD) | 41.2 (12.9) | 37.2 (13.9) | 43.7 (12.2) | 41.1 (13.0) |
